# Characterisation of the bacterial microbiota of the vagina of dairy cows and isolation of pediocin-producing *Pediococcus acidilactici*

**DOI:** 10.1186/1471-2180-13-19

**Published:** 2013-01-29

**Authors:** Yvonne Wang, Burim N Ametaj, Divakar J Ambrose, Michael G Gänzle

**Affiliations:** 1 Department of Agricultural, University of Alberta, Food and Nutritional Science, 4-10 Ag/For Centre, Edmonton, AB, T6G 2P5, Canada; 2 Alberta Agriculture and Rural Development, Agriculture Research Division, Edmonton, AB, T6H 5T6, Canada

**Keywords:** Lactic acid bacteria, Dairy cow, Metritis, Pediocin PA-1/AcH, Shiga-like toxin

## Abstract

**Background:**

Uterine infections in dairy cows lower profitability of dairy operations. Infections of the reproductive tract are related to the overgrowth of pathogenic bacteria during the first three weeks after parturition. However, alterations in the vaginal microbiota composition in the first weeks after parturition remain poorly documented.

**Results:**

In this study, bacteria isolated from the vagina of healthy pregnant, and infected postpartum cows were characterised by random amplification of polymorphic DNA (RAPD) analysis and partial 16S ribosomal RNA (rDNA) gene sequencing. Populations of bacilli and lactic acid bacteria of the genera *Enterococcus*, *Lactobacillus*, and *Pediococcus* were present in both healthy and infected cows. Infected cows had a significant increase in the vaginal enteric bacteria population which consisted mainly of *Escherichia coli*. Three *E. coli* isolates harboured the gene coding for Shiga-like-toxin (SLT) I or II. Several isolates of the *Pediococcus acidilactici* were found to produce the bacteriocin pediocin AcH/PA-1. Quantitative PCR analyses of vaginal mucus samples collected from ten metritic cows before and after parturition confirmed the presence of the Lactobacillus group (*Lactobacillus* spp., *Pediococcus* spp., *Leuconostoc* spp., and *Weissella* spp.); *Enterobacteriaceae*, *E. coli*, and bacilli. The presence of the pediocin AcH/PA-1 structural gene and SLT genes were also confirmed with qPCR.

**Conclusions:**

In conclusion, overgrowth of pathogenic bacteria, particularly *E. coli,* after parturition likely contributes to the development of metritis. Our microbiota analysis extends the information related to the composition of commensal bacteria in the bovine female reproductive tract and may facilitate the development of novel intervention strategies for prevention of uterine infections in dairy cows.

## Background

Infection of the uterus has a significant impact on the profitability of the dairy industry because of lowered reproductive efficiency, decreased milk production, and increased costs associated with treatment and culling of animals due to infertility [1-3]. Uterine infections in dairy cows are associated with predisposing factors including calving difficulty, retained placenta, compromised immune status and parity, along with the overgrowth of pathogenic microorganisms in the reproductive tract [4]. Immediately after calving, the dilated state of the cervix allows microorganisms from the environment, cow’s skin, and fecal material to enter through the vagina into the uterus and initiate inflammation of the endometrium, which is highly associated with infertility [5]. Metritis associated bacteria have been classified as pathogens, potential pathogens, or opportunistic pathogens [6,7]. Recognised uterine pathogens that are associated with severe endometrial inflammation and clinical endometritis include *Escherichia coli*, *Arcanobacterium pyogenes*, *Fusobacterium necrophorum*, *Prevotella melaninogenica* and *Proteus* species [6,7]. Williams et al. [8] considered high cell counts of *E. coli* as the basis for the onset of uterine infection.

In a healthy female reproductive tract of humans, mice, or monkeys, lactobacilli are among the predominant organisms [9-11]. Vaginal lactobacilli inhibit the growth of genitourinary pathogenic micro-organisms through mechanisms of competitive exclusion of pathogens, stimulation of the host immune system, and production of specific antibacterial compounds such as acetic and lactic acids, hydrogen peroxide, and antimicrobial peptides [12,13]. A contribution of bacteriocin production by vaginal probiotics to probiotic activity has not been demonstrated experimentally**,** but formation of the bacteriocin Abp118 by *Lactobacillus salivarius* UC118 conferred resistance to infection by *Listeria monocytogenes* in mice [14].

The microbial flora of a healthy bovine reproductive tract consists of a combination of aerobic, facultatively anaerobic, and obligately anaerobic microorganisms. Lactobacilli were found to be present in low numbers in the bovine vaginal microbiota [15]; additionally, *Enterobacteriaceae* are among the dominant populations [16]. However, alterations in the vaginal microbiota composition in the first weeks after parturition, i.e. the time during which metritis develops, remain poorly documented. The aim of our study is to characterize the vaginal microbiota of both healthy pregnant and infected post-partum cows by culture-dependent analysis. In addition, retrospective culture independent quantitative PCR (qPCR) analysis was used to characterize the vaginal microbiota of metritic cows two weeks before and two weeks calving. Isolates were studied with regards to Shiga-like toxin and pediocin production.

## Results

### Composition of microbiota in healthy and infected dairy cows: Isolation and identification of bacterial species

Analysis of the microbiota of the reproductive tract of dairy cows was initially based on a qualitative, culture-dependent approach. Bacterial isolates were obtained from healthy, pre-partum animals (n = 7) or metritic, post-partum animals (n = 8). Clonal isolates were eliminated by RAPD-PCR analysis and isolates differing in their origin, RAPD profile, or colony morphology were identified on the basis of the sequence of approximately 1400 bp of the 16S rRNA genes. Strain identification to species level was based on 97% or greater sequence homology to type strains. Strains of the species *E. coli* could not be identified on the basis of 16S rRNA sequences alone because of the high homology of rDNA sequences to closely-related species such as *Shigella* spp. and *Escherichia fergusonii*. Classification of all *E. coli* strains was verified with species-specific PCR and API-20E test strips. The biochemical characteristics of isolates matched properties of *E. coli* (99.8%) in the API-20E database. The identity of thirty isolates and their origin is listed in Table 1.

**Table 1:** Qualitative characterization of the vaginal microbiota of dairy cows

**Animal #**	**FUA #**	**Identified Species**	**% Identity to Type Strain**^**(a)**^	**Shiga -like Toxin Gene**	**Pediocin Immunity Gene**
2102 (Healthy)	3086	*Staphylococcus epidermidis*	0.990	n.d.	n.d.
	3087	*Staphylococcus epidermidis*	0.991	n.d.	n.d.
	3088	*Staphylococcus warneri*	0.985	n.d.	n.d.
	3089	*Lactobacillus sakei*	0.986	n.d.	n.d.
2151 (Healthy)	1167	*Proteus mirabilis*	0.995	n.d.	n.d.
2363 (Healthy)	1035	*Escherichia coli*	0.980 (*Shigella flexneri*)	-	n.d.
	1037	*Escherichia coli*	0.930	SLT-II	n.d.
	3137	*Pediococcus acidilactici*	0.990	n.d.	**+**
	3140	*Pediococcus acidilactici*	1.000	n.d.	**+**
	3141	*Enterococcus faecalis*	0.990	n.d.	n.d.
	3226	*Pediococcus acidilactici*	0.990	n.d.	-
2367 (Healthy)	3136	*Staphylococcus warneri*	0.993	n.d.	n.d.
2374 (Healthy)	1062	*Escherichia coli*	0.976 (*Shigella flexneri*)	SLT-II	n.d.
	2027	*Bacillus licheniformis*	0.982	n.d.	n.d.
	2028	*Bacillus licheniformis*	0.978	n.d.	n.d.
	3251	*Streptococcus pluranimalium*	0.990	n.d.	n.d.
2409 (Healthy)	1046	*Escherichia coli*	0.978 (*Shigella flexneri*)	-	n.d.
	3135	*Staphylococcus hominis* subsp. *hominis*	0.991	n.d.	n.d.
2426 (Healthy)	2023	*Bacillus altitudinis*	0.998	n.d.	n.d.
	2024	*Bacillus pumilus*	0.981	n.d.	n.d.
*2211-A (Infected)	1036	*Escherichia coli*	0.981(*Shigella flexneri*)	-	n.d.
	3139	*Enterococcus faecalis*	0.980	n.d.	n.d.
*2211-B (Infected)	1174	*Escherichia coli*	0.980	-	n.d.
	1176	*Escherichia coli*	0.980	-	n.d.
	2044	*Bacillus licheniformis*	0.998	n.d.	n.d.
	2045	*Bacillus galactosidilyticus*	0.990	n.d.	n.d.
	2049	*Bacillus oleronius*	0.990	n.d.	n.d.
	2052	*Rummeliibacillus pycnus*	0.970	n.d.	n.d.
2312 (Infected)	2039	*Bacillus licheniformis*	0.982	n.d.	n.d.
	2047	*Lysinibacillus fusiformis*	0.970	n.d.	n.d.
	2048	*Sporosarcina contaminans*	0.980	n.d.	n.d.
	2050	*Streptococcus thoraltensis*	0.990	n.d.	n.d.
	2051	*Rummeliibacillus pycnus*	0.970	n.d.	n.d.
	3308	*Lactobacillus mucosae*	0.996	n.d.	n.d.
2373 (Infected)	1063	*Escherichia coli*	0.987 (*Shigella flexneri / Escherichia fergusonii*)	-	n.d.
2429 (Infected)	3227	*Staphylococcus warneri*	0.990	n.d.	n.d.
	3138	*Pediococcus acidilactici*	0.990	n.d.	+
2435 (Infected)	1049	*Escherichia coli*	0.980 (*Shigella flexneri* / *Escherichia fergusonii*)	-	n.d.
2436 (Infected)	1070	*Escherichia coli*	0.973 (*Escherichia fergusonii*)	-	n.d.
2507 (Infected)	1064	*Escherichia coli*	0.960 (*Shigella flexneri*)	SLT-I	n.d.
	3180	*Streptococcus pluranimalium*	0.990	n.d.	n.d.
	2029	*Bacillus licheniformis*	0.995	n.d.	n.d.

^(a)^ % identity of partial 16S rDNA to type strain or closest relative; **+**: positive PCR results; **-**: negative PCR results; n.d.: data not determined

*Cow #2211-A and 2211-B represent two different animals that were assigned the same number at different times.

Healthy, pregnant animals and those diagnosed with post partum uterine infections at the time of sampling are indicated in brackets.

**Figure 1:**
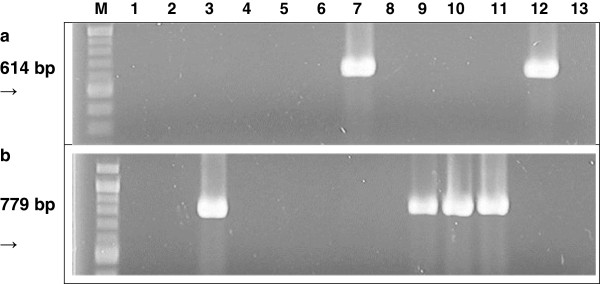
**PCR-based detection of shiga-like toxins.** Panel **a**. PCR-based detection of shiga-like toxin I (SLT-I)-producing *E. coli* FUA1064 (lane 7). DNA extracted from *E. coli* O157:H7 ATCC43890 was used as positive control for SLT-I (lane 12). Panel **b**. PCR based detection of SLT-II-producing *E. coli* FUA1037 (lane 3), and *E. coli* FUA1062 (lanes 9 and 10). DNA extracted from *E. coli* O157:H7 ATCC 43889 was used as positive control for SLT-II (lane 11).

Bacilli, staphylococci, and lactic acid bacteria of the genera *Enterococcus*, *Lactobacillus*, and *Pediococcus* were present in both healthy and infected cows. *Escherichia coli* were also frequently isolated, particularly from infected animals. Isolates were screened for the presence of SLT-I and SLT-II genes, sample results for their PCR detection in *E. coli* isolates are shown in Figure 1a and Figure 1b, respectively. *E. coli* FUA1064 isolated from cow #2507 harboured the SLT-I gene, while *E. coli* FUA1037 and FUA1062, isolated from cow #2373 and #2374, respectively harboured the SLT-II gene (Table 1).

### Pediocin production

**Figure 2:**
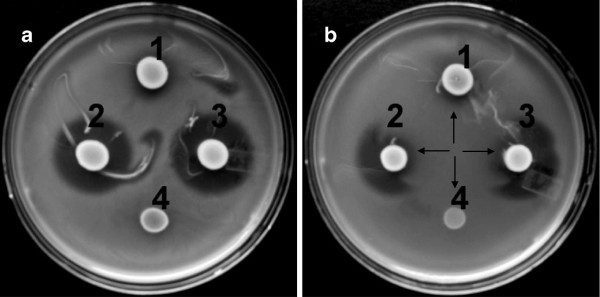
**Deferred inhibition assay for bacteriocin production.** Test strains were grown on mMRS and overlayered with Enterococcus faecalis FUA3141, which was as an indicator strain. Panel **a**, no addition of proteinase; panel **b**, addition of *proteinase* K adjacent to colonies of test strains. Arrows indicate the site of proteinase K application. The following test strains were used, 1, *Ped. acidilactici* FUA3138; 2, *Ped. acidilactici* FUA3072; 3, *Ped. acidilactici* FUA3140; 4, *Lact. sakei* FUA3089. Similar results were observed with *Listeria innocua* ATCC33090 used as an indicator strain (data not shown). The indicator strains of *E. coli* FUA1036, FUA1063 and FUA1064 were also used but no inhibition was observed (data not shown).

PCR screening revealed that *Ped. acidilactici* FUA3137, FUA3140, and FUA3138 harboured the pediocin AcH/PA-1 immunity gene (Table 1). Pediocin production was investigated for selected isolates via deferred inhibition assays. *Ped. acidilactici* FUA3138 and FUA3140 produced inhibition zones against *Enterococcus faecalis* FUA3141 (Figure 2a)*.* Inhibition zones of comparable diameter were observed with *L. innocua* (data not shown). Further tests with proteinase K verified that the antimicrobial agent is a protein (Figure 2b). Other vaginal isolates including *E. coli* FUA1036, FUA1063, and FUA1064 were also used as indicator strains but no inhibition was observed (data not shown).

### Quantification of bacterial groups, SLT and pediocin structural genes

**Figure 3:**
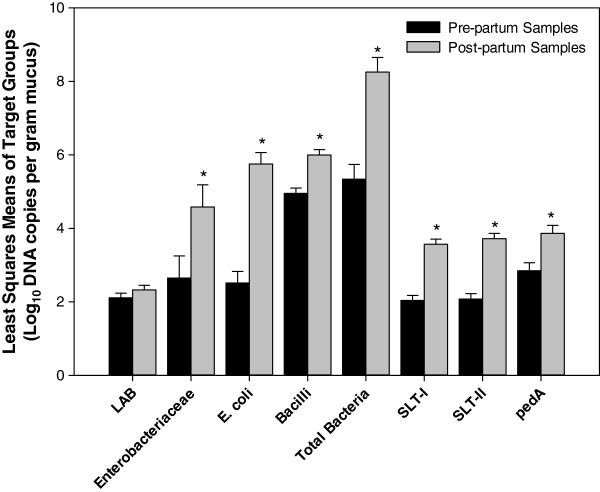
**Differences in least squares means of log rDNA or DNA copy numbers of target groups**. Vaginal mucus was sampled from ten animals before and after calving, and bacterial rDNA, shiga-like-toxin genes, and the pediocin structural gene were quantified by qPCR. The figure depicts the differences in least squares means of the target groups. Statistically significant differences between prepartum and postpartum periods were observed in all groups (as indicated by *) except for the lactic acid bacteria group.

The DNA concentration of most samples did not allow amplification with HDA primers; PCR products could be obtained only for two samples (data not shown). Sequencing of the PCR products from these animals (#2373 and #2409) confirmed that bacteria present in the bovine vagina of these two animals were accounted for by culturing (data not shown). Subsequently, quantitative PCR was employed as sensitive and quantitative tool for culture-independent analysis of the composition of vaginal microbiota before and after parturition. Primers were selected to quantify bacterial groups isolated from healthy, pre-partum or postpartum animals, as well as SLT genes and the pediocin structural gene (pedA) (Table 1). Fourty animals were sampled two weeks pre-partum and two weeks post-partum; of these, ten animals that developed metritis post-partum were selected for DNA isolation and analysis by qPCR. To account for the large individual differences in the vaginal microbiota of different animals, results were calculated as differences (post-partum – pre-partum) between the least square means of log rDNA or DNA copy numbers for each target group (Figure 3). Copy number of 16S rDNA from *Enterococcus* spp. and *Staphylococcus* spp. were below the detection limit of 10^2^ copy numbers / g (data not shown). The number of rDNA copies of the *Lactobacillus* group was relatively stable in the observation period. In all other cases, the postpartum gene copy values are higher than the prepartum values. The pediocin structural gene was consistently detected in low numbers. Approximately a 3 log difference between the total bacteria values was observed. This increase was predominantly attributable to increased numbers of *E. coli* and *Enterobacteriaceae*. *E. coli* increased on average by more than 3 log. Genes coding for SLT-I and SLT-II increased by less than 2 log.

## Discussion

This study provides a comparison of the vaginal microbiota of healthy, pregnant dairy cows, and infected postpartum cows. In contrast to the stable commensal microbiota observed in humans and other mammals [9-11], total bacterial numbers in vaginal mucus were low and the composition of the bovine vaginal microbiota on species level was highly variable. Bacteria found within the microbiota are thus likely to be contaminants from the environment (*Bacillus* spp.), the cow’s skin (*Staphylococcus* spp.), or faecal material (*E. coli,* lactic acid bacteria), rather than representing a stable flora autochthonous to the reproductive tract. The lack of a competitive commensal vaginal microbiota may contribute to the susceptibility of dairy cows to bacterial overgrowth and metritis after parturition [8,17]. Indeed, quantitative PCR demonstrated a substantial increase of bacterial numbers, particularly of *Enterobacteriaceae* and *E. coli*, in infected cows after parturition compared to samples from the same animals obtained pre-partum.

Overall, our data indicated that vaginal bacterial flora in cows affected by metritis was dominated by strains of *E. coli*, supporting previous observations [17]. This study extends previous results [15,16] by documenting changes of the vaginal microbiota in individual animals in the first two weeks after calving. Both the *Enterobacteriaceae* and *E. coli* showed marked increase in mucus samples collected from infected postpartum cows. The amplification of *Shigella* rDNA with *E. coli* species-specific primers is not surprising because *Shigella* spp. and *E. coli* are indistinguishable on the basis of rDNA sequences [18]. In keeping with the recognition of *Shigella* spp. as human-adapted pathovar of *E. coli*, all isolates were identified as *E. coli* by biochemical tests. Culture-based analysis and qPCR demonstrated presence of shiga-like-toxin producing *E. coli* (STEC) in both healthy and infected animals. Three out of eleven *E. coli* isolates were found to carry genes coding for SLT-1 or SLT-II. Moreover, SLT-genes were consistently detected by qPCR in samples from metritic cows; STEC accounted for about 1 – 10% of the total *E. coli* population. SLT production causes diarrhoea in calves [19], but the role of STEC in the pathogenesis of metritis in adult animals warrants further clarification.

Bacilli are present in the environment and they frequently contaminate the bovine uterine lumen [20]. However, pediococci have not yet been described as part of the bovine vaginal microbiota. The genus *Pediococcus* is closely related to the genus *Lactobacillus*. Pediococci produce antimicrobial compounds such as organic acids, hydrogen peroxide, and antimicrobial peptides such as pediocin AcH/PA-1 [21]. *Ped. acidilactici* is a food fermenting organism [21] but was also isolated from the gastrointestinal tract of poultry, ducks, and sheep[22-24]. Pediocin AcH/PA-1 producing strains have been isolated from human infant faeces [25].

The synthesis of pediocin AcH/PA-1 was initially described for the strains *Ped. acidilactici* PAC1.0 and *Ped. acidilactici* H, but synthesis has also been observed in other *Ped. acidilactici* strains as well as *Lactobacillus plantarum* WHE92, *Pediococcus parvulus* ATO34, and ATO77 [26-28]. Pediocin AcH/PA-1 production is a plasmid-borne trait [29]. The pediocin AcH/PA-1 operon consists of pediocin AcH/PA-1 gene (*pedA/papA*), a specific immunity gene (*papB*)*,* and genes responsible for processing and secretion (*papC* and *papD*) [30]. In keeping with prior reports on pediocin activity [31], pediocin was not active against *E. coli*, the dominant organisms in the vaginal microbiota of infected animals. Pediocin producing isolates characterized in this study harboured the pediocin AcH/PA-1 operon, and qPCR analysis consistently detected the operon in both prepartum and postpartum vaginal samples.

Bacteriocin formation is increasingly recognized as an important trait of probiotic cultures [32]. Studies on the isolation of bacteriocin-producing lactic acid bacteria from the human vagina demonstrated their antimicrobial activities against human vaginal pathogens [33,34]. Bacteriocin-producing *Lactobacillus* strains inhibited vaginal pathogens including *Gardnerella vaginalis* and *Pseudomonas aeroginosa*[35]. Although bovine vaginal microbiota have much lower total cell counts and lactobacilli populations in comparison to the human vaginal microbiota [16,36], bacteriocin such as pediocin may influence the microbial ecology in the reproductive tract of dairy cattle if bacteriocin-producing lactic acid bacteria are administered in high numbers.

## Conclusions

In conclusion, culture-dependent analysis of the bacterial vaginal microbiota of dairy cows, supported by qPCR analysis, allowed the characterization of the bovine vaginal microbiota of healthy pregnant and infected postpartum cows. Identification of pediocin-producing pediococci in the bovine vaginal microbiota may allow the development of novel prophylactic interventions against metritis by application of bacteriocin-producing probiotic bacteria into the vaginal tract of dairy cows.

## Methods

### Animals

In a first experiment, fifteen lactating Holstein dairy cows were used to characterize the vaginal microbiota of healthy pregnant and metritic postpartum cows. In a second experiment, ten animals were selected to characterize the vaginal microbiota of metritic cows two weeks before calving and two weeks after calving. Samples from these ten animals were selected retrospectively after diagnosis of metritis among a group of 40 dairy cows. All animals were maintained at the Dairy Research and Technology Centre of the University of Alberta. Metritis or uterine infections were diagnosed on the basis of criteria established by Sheldon et al. [1]. Primarily, cows with watery reddish-brown, purulent, or mucopurulent discharges with fetid odour were considered to have metritis. Rectal temperatures of 39.5°C or higher and impaired general condition as expressed in a lowered feed intake or milk production were also taken into consideration for diagnosis. Ethics approval was obtained from the Animal care and Use Committee for Livestock of the Faculty of Agricultural, Life and Environmental Sciences (University of Alberta protocol #A5070-01).

### Samples

For culture-dependent analyses in experiment 1, vaginal swab samples were obtained from seven healthy pregnant cows and eight infected post-partum cows. The vulvar area was thoroughly cleaned with water and then disinfected with 30% (vol/vol) iodine solution (Iosan, WestAgro, Saint Laurent, Canada) prior to sampling. A stainless steel vaginal speculum was gently inserted into the vagina, opened, and a long-handled sterile cotton swab was introduced to obtain a sample from the anterolateral vaginal wall. Each sample was collected in 4 mL of 0.1% (w/v) sterile peptone water with 0.85% (w/v) NaCl and 0.05% (w/v) L-cysteine-HCl x H_2_O. The cotton swab was moistened by immersion in the peptone water immediately before sampling. Owing to the low amount of mucus retrieved from healthy, pregnant cows, the weight of the mucus recovered was not recorded.

For culture-independent analyses in experiment 2, vaginal mucus samples were collected using syringes fitted with an approximately 30 cm long collection tube without the use of a vaginal speculum. The weight of mucus in each sample was determined by recording the total weight of each sample collection tube with 1 ml of peptone water before and after each mucus sample was collected. All samples were stored at temperatures between −20°C to −80°C.

### Isolation of microorganisms

Ten-fold serially diluted samples were plated on Reinforced Clostridial Medium (RCM) with 5% animal blood, Endo agar (Difco, Sparks, USA), and modified MRS (mMRS) agar [37]. Representative colonies from each type of plates and colony morphology were purified by repeated streak-plating until a uniform colony morphology was obtained. Isolates from mMRS and RCM with blood were streaked on mMRS agars whereas isolates from Endo plates were streaked on Luria Bertani (LB) agars. Frozen stock cultures of each isolate were prepared from a single colony and stored in 60% glycerol at −70°C.

### General molecular techniques

General DNA manipulations and agarose gel electrophoresis were performed as described by Sambrook *et al.*[38]. Chromosomal DNA of isolated strains was extracted from 1 ml cultures using a DNeasy® Blood and Tissue Kit (Qiagen, Mississauga, Canada). Unless otherwise stated, PCR amplifications were performed in GeneAmp® PCR System 9700 (Applied Biosystems, Streetsville, Canada) by using Taq DNA polymerase and deoxynucleoside triphosphates (Invitrogen, Burlington, Canada). The PCR products were purified using the QIAquick PCR purification kit (Qiagen).

### Random amplified polymorphic DNA-PCR (RAPD-PCR) analysis

RAPD typing was used to identify clonal isolates. Isolates with the same origin, the same colony morphology, and identical RAPD patterns were considered clonal isolates. DNA template was isolated as described above. DAF4 primer was used to generate RAPD patterns for isolates from Endo agar and M13V primer was used for RAPD typing of all other strains (Table 2). The reaction mixture contained 10 μL of 5x Green GoTaq® Reaction Buffer (Promega, San Luis Obispo, USA), 3 μL of 25 mM MgCl_2_ (Promega), 150 pmol primer (Table 2), 1 μL of 10 mmol L^-1^ dNTP (Invitrogen, Burlington, Canada), 1.5 U GoTaq® DNA Polymerase (Promega), and 1 μL of template DNA suspension or autoclaved water filtered with Milli-Q water purification system as the negative control (Millipore Corporation, Bedford, Massachusetts, United States). The PCR program comprised of an initial denaturation step at 94°C for 3 minutes, followed by 5 cycles of denaturation, annealing and extension steps at 94°C for 3 minutes, 35°C for 5 minutes, and 72°C for 5 minutes. An additional 32 cycles of denaturation, annealing and extension steps were also performed at 94°C for 1 minute, 35°C for 2 minutes, 72°C for 3 minutes, followed by a final extension step at 72°C for 7 minutes. RAPD PCR products were electrophoresed in a 1.5% agarose gel with 0.5x TBE buffer (45 mmol L^-1^ Tris base, 45 mmol L^-1^ boric acid, 1 mM EDTA, pH 8.0); isolates from the same animal were electrophoresed on the same gel. A 2-log molecular size marker (New England Biolabs, Pickering, Canada) was included on all gels.

**Table 2:** Primers used in the study

**Target/Specificity**	**Primer/Probe Sequence (5’ → 3’)**	**Annealing Temperature (°C)**	**Reference**
†*Lactobacillus* –*Pediococcus-Leuconostoc-Weissella* (*Lactobacillus* group) (341 bp)	**Lac1:** AGC AGT AGG GAA TCT TCC A	62	[39,40]
	**Lab667r:** CAC CGC TAC ACA TGG AG		
†*Enterococcus* spp.(144 bp)	**Ent-F:** CCC TTA TTG TTA GTT GCC ATC ATT	60	[41]
	**Ent-R:** ACT CGT TGT ACT TCC CAT TGT		
†*Enterobacteriaceae* (195 bp)	**Enterobac-F:** CAT TGA CGT TAC CCG CAG AAG AAG C	63	[42]
	**Enterobac-R:** CTC TAC GAG ACT CAA GCT TGC		
†*Staphylococcus* spp. (370 bp)	**TStaG422:** GGC CGT GTT GAA CGT GGT CAA ATC	55	[43]
	**TStaG765:** TIA CCA TTT CAG TAC CTT CTG GTA A		
†*Bacillus* spp. (995 bp)	**BacF:** GGGAAACCGGGGCTAATACCGGAT	55	[44]
	**BacR:** GTC ACC TTA GAG TGC CC		
†*E. coli* (544 bp)	**ECP79F:** GAA GCT TGC TTC TTT GCT	54	[45]
	**ECP620R:** GAG CCC GGG GAT TTC ACA T		
†SLT-I (614 bp)	**VT1 (SLTI-F):** ACA CTG GAT GAT CTC AGT GG	55	[44]
	**VT2 (SLTI-R):** CTG AAT CCC CCT CCA TTA TG		
†SLT-II (779 bp)	**VT3 (SLTII-F):** CCA TGA CAA CGG ACA GCA GTT	55	
	**VT4 (SLTII-R):** CCT GTC AAC TGA GCA CTT T		
16S rDNA Sequencing	**616V:** AGA GTT TGA TYM TGG CTC	52	[46]
(~1500 bp)	**630R:** AAG GAG GTG GAT CCA RCC		
	CAKAAAGGAGGTGGATCC		
Random Primer for RAPD	**DAF4:** CGG CAG CGC C	35	[47]
	**M13V:** GTT TTC CCA GTC ACG ACG TTG	35	[48]
Universal Primers	**HDA1:** ACT CCT ACG GGA GGC AGC AG	52	[49]
	**HDA2:** GTA TTA CCG CGG CTG CTG GCA		
	**HDA1 + GC:** CGC CCG GGG CGC GCC CCG GGC GGG GCG GGG GGC ACG GGG GGA CTC CTA CGG GAG GCA GCA G		
TA Cloning	**M13Forward (−20):** GTA AAA CGA CGG CCA G	55	[50]
	**M13Reverse:** CAG GAA ACA GCT ATG AC		
†Pediocin Structural Gene pedA (100 bp)	**pedA2RTF:** GGC CAA TAT CAT TGG TGG TA	60	[25]
	**pedA2RTR:** ATT GAT TAT GCA AGT GGT AGC C		
	**TqM-pedA:** FAM-ACT TGT GGC AAA CAT TCC TGC		
	TCT GTT GA-TAMRA		
†Total Bacteria (727 bp)	**TotalBac-F785:** GGA TTA GAT ACC CTG GTA GTC	52	[51-53]
	**TotalBac-R1512r:** TAC CTT GTT ACG ACT T		
	**TaqMan 1400r Probe**: 6-FAM-TGA CGG GCG GTG TGT ACA AGG C-TAMRA		

^†^ All dagger-marked primer pairs were used in the preparation of standards and qPCR analyses.

### Partial 16S ribosomal rRNA gene amplification and sequencing

Isolates differing in origin or RAPD pattern were identified by partial sequencing of 16S rRNA genes. PCR reaction was performed in a master mix with a final volume of 50 μL containing 1.5 U Taq DNA Polymerase (Invitrogen), 5 μL of 10X PCR Reaction Buffer (Invitrogen), 1.5 μL of 25 mmol L^-1^ MgCl_2_ (Invitrogen), 25 pmol of universal bacterial primers 616V and 630R (Table 2), 1 μL of 10 mmol L^-1^ dNTP, and 1 μL of template DNA. PCR product was electrophoresed in 1.0% (w/v) agarose gel, with a 2-log ladder (New England Biolabs). All sequencing data were obtained from sequencing services provided by Macrogen (Rockville, USA). The 16S rRNA gene sequences of isolates were compared with 16S rRNA gene sequences of type strains in the Ribosomal Project Database Project II (RDP-II; Michigan State University, East Lansing, USA, http://rdp.cme.msu.edu).

### Identification of *E. coli* with species-specific PCR and API 20E test system

PCR amplification of the hypervariable regions of the *E. coli* 16S rRNA gene used primers described by Sabat *et al.*[45]. The PCR reaction mix (final volume 50 μL) consisted of 1.25 U Taq DNA Polymerase (Invitrogen), 5 μL of 10X PCR Reaction Buffer (Invitrogen), 1.5 μL of 25 mmol L^-1^ MgCl_2_ (Invitrogen), 100 pmol of ECP79F and ECP620R (Table 2), 1 μL of 10 mmol L^-1^ dNTP, and 1.5 μL of template DNA. Reference strains used as positive and negative controls are listed in Table 3. The API 20E test system (bioMérieux, Saint Laurent, Canada) was used to confirm identification to the species level. PCR-based detection of Shiga-like toxin producing *E. coli* (STEC) was conducted with 50 μL reaction mixes that contained 1.25 U Taq DNA Polymerase (Invitrogen), 5 μL of 10X PCR Reaction Buffer (Invitrogen), 1.5 μL of 25 mmol L^-1^ MgCl_2_ (Invitrogen), 1 μL of 10 mmol L^-1^ dNTP (Invitrogen), 25 pmol SLTI-F and SLTI-R (Table 2), or 25 pmol SLTII-F and 25 pmol SLTII-R. Positive controls are listed in Table 3.

**Table 3:** Reference strains used in the study

**Strain**	**Description**
*Lactobacillus plantarum* FUA3099	Positive control for RAPD with M13V primer
*Shigella boydii* ATCC4388	Negative control for species specific PCR of *E. coli* 16S rRNA gene
*Shigella dysenteriae* ATCC188	
*Shigella flexneri* ATCC62	
*E. coli* O157:H7 ATCC43888	Positive control for species specific PCR of *E. coli* 16S rRNA gene
*E. coli* O157:H7 ATCC43889	SLT-II positive control
*E. coli* O157:H7 ATCC43890	SLT-I positive control
*Pediococcus acidilactici* FUA3072	Bacteriocin-producing strain expressing the pediocin AcH/PA-1 operon
*Listeria innocua* ATCC33090	Indicator strains used in deferred inhibition assay for bacteriocins detection

### Detection of bacteriocin production by *Lactobacillus* spp. and *Pediococcus* spp

*Lactobacillus* species and *Pediococcus* species were initially screened for production of pediocin AcH by PCR amplification of the pediocin AcH immunity gene. The gene amplification was performed with 50 μL reaction mixes that contained 1.5 U Taq DNA polymerase (Invitrogen), 5 μL of 10X PCR reaction buffer (Invitrogen), 1.5 μL of 25 mM MgCl_2_ (Invitrogen), 1 μL of 10 mM dNTP (Invitrogen), 2 μL of template DNA, 25 pmol of primers Pediocin-for (TCA ATA ATG GAG CTA TGG) and Pediocin-rev (ACC AGT CTC CAG AAT ATC TAA). Bacteriocin production by lactic acid bacteria was determined with bacteriocins screening medium as described [54]. Overnight cultures of test strains were prepared in MRS broth that contained 2 g L^-1^ glucose. Test strains used in this study included *Lactobacillus sakei* FUA3089 as well as *Ped. acidilactici* FUA3138 and FUA3140. MRS plates with 2 g glucose L^-1^ were spotted with 3 μL of each overnight culture and the plates were incubated overnight under anaerobic conditions at 37°C. *Ped. acidilactici* FUA3072 was used as reference strain. Bacteriocin formation of this strain was previously characterized by sequencing of the pediocin operon, quantification of the expression of genes of the pediocin operon, and deferred inhibition assay (data not shown).

Cultures of indicator strains (Table 3) grown in overnight MRS broth with 2 g L^-1^ glucose were used to inoculate MRS soft agar at an inoculation level of 1% and the soft agar was overlayered over the MRS plates with test strains. Indicator strains included *E. coli* FUA1036, *E. coli* FUA1063, *E. coli* FUA1064, *Listeria innocua* ATCC33090, and *Enterococcus facaelis* FUA3141.

The deferred inhibition assay was repeated with the addition of 20 g L^-1^ proteinase K in 100 mmol L^-1^ Tris-Cl, pH 8.5, which was spotted adjacent to test strain colonies and plates were incubated for four hours at 55°C to maximize proteinase activity before overlayering was conducted.

### Identification of library clones via sequencing

PCR-DGGE analysis was initially carried out characterise bovine vaginal microbiota by a culture-independent approach. The DNA concentration of samples from healthy cows, however, was below the detection limit of PCR-DGGE analysis and DGGE patterns could be obtained only for two samples from animals #2373 #2409 (data not shown). Total bacterial DNA was isolated from these two vaginal swab samples via both phenol chloroform extraction and Wizard MagneSil® Tfx™ System (Promega). Nested PCR was conducted to maximize DNA amplification by amplifying with 616V and 630R primers prior to amplification with HDA primers (Table 2). PCR products that were amplified with HDA primers were cloned into a pCR 2.1-TOPO vector using the TOPO TA Cloning® Kit (Invitrogen) according to manufacturer’s instructions. The Promega’s Wizard® Plus SV A clone library was constructed using PCR products that were amplified with HDA primers, which were then cloned into a pCR 2.1-TOPO vector, using the TOPO TA Cloning® Kit (Invitrogen) according to manufacturer’s instructions. The Promega’s Wizard® Plus SV Minipreps DNA Purification System was used for plasmid isolation. To confirm the cloning of the inserts, sequencing of the amplified insert was performed according to the Invitrogen TOPO TA Cloning® Kit manual.

### Quantitative PCR

Quantitative PCR was conducted with vaginal mucus samples collected from ten cows, using syringes fitted with an approximately 30 cm long collection tube. Samples from 10 animals that developed metritis after calving were randomly selected from samples of a larger cohort of animals. Total bacterial DNA was extracted using the Wizard MagneSil® Tfx™ System (Promega) and DNA concentrations were measured using the NanoDrop spectrophotometer system ND-1000, software version 3.3.0 (Thermo Fisher Scientific Inc., Wilmington, USA). All dagger-marked primer pairs that are listed in Table 2 were used in the preparation of standards and qPCR analyses. Standards were prepared using purified PCR products, which were serially diluted ten-fold. Diluted standards (10^-3^ to 10^-8^) were used to generate standard curves. TaqMan probes were used for the pedA gene and the total bacteria qPCR experiments. In both cases, each probe was labelled with 5’-FAM and 3’-TAMRA as fluorescent reporter dye and quencher respectively. The total reaction volume was set to 25 μL, which contained 12.5 μL TaqMan Universal PCR Master Mix (Applied Biosystems), 2.5 μL of template DNA extracted from vaginal mucus and 5 μmol L^-1^ of each primer (Table 2), and 0.2 μmol L^-1^ of the TaqMan probe. SYBR green assays were used for all remaining target-group primer pairs. The total reaction was also set at 25 μL containing 12.5 μL Fast SYBR Green Master Mix (Applied Biosystems), 1 μmol L^-1^ primer, and 1 μL DNA template. Amplification conditions generally followed an initial denaturation at 95°C for 5 min for 1 cycle; 40 cycles of denaturation at 95°C for 30 sec, annealing with listed annealing temperatures in Table 2 for 1 min, and extension at 72°C for 2 min. Quantitative PCR was executed using a 7500 Fast Real-Time PCR System (Applied Biosystems, Foster City, CA, USA). Reactions were performed in triplicates in MicroAmp Fast Optical 96-well reaction plates, sealed with MicroAmp Optical Adhesive Film (Applied Biosystems).

### Statistical analysis

Results were analyzed using the general linear models procedure of SAS (Release 9.2, SAS Institute, Inc., Cary, NC, USA). The mathematical model used one animal as experimental unit and included the type of bacteria as the dependent variable and tested for differences in the least square means of log rDNA or DNA copy numbers for each target group between the two periods (i.e., pre-partum versus post-partum).

### Gene accession numbers of 16S rRNA gene sequences obtained in this study

Sequences of 16S rRNA genes of isolates obtained in this study were deposited in GenBank® with the following accession numbers: FUA3086 (GQ222397), FUA3087 (GQ222398), FUA3088 (GQ222399), FUA3089 (GQ222408), FUA1167 (GQ205673), FUA1035 (GQ222390), FUA1037 (GQ222410), FUA3137 (GQ222393), FUA3140 (GQ222392), FUA3141 (GQ222407), FUA3226 (GQ222394), FUA3136 (GQ205672), FUA1062 (GQ222401), FUA2027 (GQ205674), FUA2028 (GQ222400), FUA3251 (GQ222395), FUA1046 (GQ222387), FUA3135 (GQ222404), FUA2023 (GQ205670), FUA2024 (GQ205671), FUA1036, (GQ222389), FUA3139 (GQ222406), FUA1063 (GQ222403), FUA3227 (GQ205669), FUA3138 (GQ222409), FUA1049 (GQ222388), FUA1070 (GQ222391), FUA1064 (GQ222405), FUA3180 (GQ222402), FUA2029 (GQ222396).

## Competing interests

The authors declare that they have no competing interests.

## Authors' Contributions

YW, BA, DA and MGG designed research; DA collected samples and diagnosed metritis in post-partum animals; YW assisted with sample collections and conducted the research; YW, DA and MGG analyzed data; YW, BA, DA and MGG wrote the paper; and MGG had primary responsibility for final content. All authors read and approved the final manuscript.
